# Development of a risk stratification tool for rapidly progressive diabetic retinopathy in type 2 diabetes

**DOI:** 10.3389/fendo.2026.1837957

**Published:** 2026-06-19

**Authors:** Aiping Gu, Zhichao Yan, Yi Wu, Yanying Li, Renlong Liang, Xiaodi Tang, Mengke Li

**Affiliations:** Department of Ophthalmology, The Affiliated Guangdong Second Provincial General Hospital of Jinan University, Guangzhou, Guangdong, China

**Keywords:** machine learning, nomogram, prediction model, rapidly progressive diabetic retinopathy, risk stratification, type 2 diabetes mellitus

## Abstract

**Background:**

The progression of rapidly progressive diabetic retinopathy (PDR) in type 2 diabetes mellitus (T2DM) is characterized by substantial inter-individual variability. To develop and validate a nomogram for individualized risk prediction and stratification of rapidly progressive PDR in T2DM by incorporating diabetes duration, glycated hemoglobin (HbA1c), 24-hour urinary protein quantification, growth differentiation factor 15 (GDF15), Diabetic Retinopathy Severity Scale (DRSS) grade, and foveal avascular zone area.

**Methods:**

This retrospective study enrolled 342 patients with T2DM (1999 WHO criteria), randomly assigned to training (n=240) and validation (n=102) sets (7:3 ratio). Baseline demographic, clinical, metabolic, renal, inflammatory biomarker, and ophthalmic imaging data were collected. Predictive variables were selected via univariate analysis and least absolute shrinkage and selection operator (LASSO) regression. Independent predictors identified by multivariable logistic regression were incorporated into a nomogram. For comparison, Random Forest, multivariable logistic regression, and Gradient Boosting Machine models were also developed. Model performance was assessed using the area under the receiver operating characteristic curve (AUC-ROC), calibration curves, and decision curve analysis (DCA).

**Results:**

Univariate analysis identified six significant factors (all *P* < 0.05): diabetes duration, HbA1c, 24-hour urinary protein quantification, GDF15, DRSS grade, and foveal avascular zone area. LASSO regression retained all six, and multivariable logistic regression confirmed them as independent risk factors for rapidly progressive DR in T2DM (all *P* < 0.05). Three machine learning models were constructed. The Random Forest model achieved the numerically highest validation AUC (0.780) compared with Gradient Boosting Machine (0.741) and multivariable logistic regression (0.698), though the DeLong test showed no statistically significant difference between Random Forest and Gradient Boosting Machine (*P* = 0.38). Calibration curves showed good consistency between predicted and observed probabilities. DCA indicated high clinical net benefit of the model at 0.1-0.8 threshold probability vs other models and extreme strategies.

**Conclusion:**

A novel risk prediction model for rapidly progressive PDR in T2DM was developed and validated by integrating multidimensional parameters. Demonstrating favorable discrimination, calibration, and clinical utility, this model provides a promising tool for early identification of high-risk individuals and optimization of personalized intervention strategies.

## Introduction

Type 2 diabetes mellitus (T2DM) is a highly prevalent chronic metabolic disease globally, with its microvascular complications severely impacting patients’ quality of life and survival prognosis (1). Diabetic retinopathy (DR) is one of the most common microvascular complications of T2DM. As a severe subtype, rapidly progressive DR can lead to the destruction of the retinal microvascular structure, a sharp decline in visual acuity, and even blindness within a short period, establishing it as a leading cause of visual impairment in adults (2). Studies have shown significant inter-individual variability in the onset and progression of rapidly progressive DR. Currently, a reliable tool to accurately identify high-risk individuals in clinical practice is lacking, causing some patients to miss the optimal window for early intervention and ultimately resulting in irreversible visual damage (3).

The pathological mechanism of rapidly progressive DR is complex, influenced by a combination of multidimensional factors, including metabolic control, renal impairment, inflammatory stress, and baseline retinal structure (4). Diabetes duration and glycated hemoglobin (HbA1c) are classic indicators of metabolic control; chronic hyperglycemic toxicity is the core driver of microvascular damage, with elevated levels strongly correlating with an increased risk of DR progression (5). As a key marker of renal impairment, 24-hour urinary protein excretion indicates the coexistence and mutual exacerbation of diabetic nephropathy and DR, serving as a critical early warning sign for rapid DR progression (6). In recent years, inflammation-related markers such as growth differentiation factor 15 (GDF15) have been implicated in metabolic stress and endothelial dysfunction, potentially accelerating DR progression by intensifying microvascular inflammation (7). Concurrently, the baseline severity of retinal lesions (DRSS grade) and alterations in the macular microvasculature (foveal avascular zone area) directly reflect retinal ischemia and hypoxia, acting as crucial imaging predictors for the progression to proliferative DR (8). However, the comprehensive impact of these clinical, laboratory, and imaging indicators on rapidly progressive DR is complex, and a precise predictive model integrating such multidimensional information is currently unavailable.

Machine learning algorithms can efficiently process high-dimensional clinical data and capture potential non-linear relationships and interactions among variables, offering unique advantages in developing disease risk prediction models (9). Therefore, this study aims to systematically integrate multidimensional data, including clinical characteristics, laboratory parameters, and retinal imaging metrics from patients with T2DM (10). By employing machine learning methods, we intend to develop and validate a risk stratification model for predicting rapidly progressive DR in T2DM patients, thereby providing a quantitative basis for clinical decision-making to facilitate early identification of high-risk individuals and optimize personalized intervention strategies.

## Methods

### Study population

A total of 342 eligible patients with T2DM were retrospectively enrolled and randomly divided into a training set (240 patients) and a validation set (102 patients) at a 7:3 ratio. The inclusion criteria were: (1) diagnosis of T2DM according to the 1999 World Health Organization (WHO) diagnostic criteria (11); (2) age ≥ 18 years; (3) complete fundus examination data available at baseline and during follow-up to definitively determine the occurrence of rapidly progressive DR; and (4) complete baseline clinical, laboratory, and retinal imaging assessments. The exclusion criteria were: (1) diagnosis of type 1 diabetes mellitus or other specific types of diabetes; (2) presence of other ocular diseases, such as glaucoma or age-related macular degeneration, that could inter forestere with the evaluation of retinopathy; (3) severe dysfunction of vital organs (e.g., heart, liver, kidney) precluding the completion of follow-up; and (4) missing clinical or imaging data preventing inclusion in subsequent analyses.

### Data collection

Baseline multidimensional information was systematically collected from all patients via the electronic medical record system and clinical database as candidate predictors for model construction:

#### Demographics and clinical characteristics

Age, sex, diabetes duration, systolic blood pressure, body mass index, history of hypertension, smoking history, and type of glucose-lowering medication.

#### Glucose metabolism and renal function parameters

HbA1c, 24-hour urinary protein excretion, estimated glomerular filtration rate (eGFR), and cystatin C.

#### Inflammation and metabolism-related biomarkers

Low-density lipoprotein cholesterol, serum magnesium, plasma endothelin-1, β-CTX, GDF15, Renin, and Plexin B2 (12).

#### Retinal imaging indicators

DRSS grade (no/mild NPDR, moderate NPDR, severe NPDR/PDR) and foveal avascular zone area.

### Outcome definition

The primary outcome of this study was rapidly progressive DR, defined as: a ≥2-step increase in the DRSS grade from baseline during follow-up, progression to proliferative DR (PDR), or the development of vision-threatening diabetic macular edema (13). All outcomes were independently assessed by two trained ophthalmologists, with any disagreement resolved by a third senior ophthalmologist. Based on the outcome assessment, patients were classified into the rapid progression group or the non-rapid progression group.

### Statistical analysis

Data analysis was performed using SPSS 26.0, R 4.2.3, and Python 3.8.5. For continuous variables, normally distributed data were presented as mean ± standard deviation (x̄ ± s) and compared between groups using the independent samples t-test; non-normally distributed data were presented as median (interquartile range) [M (P25, P75)] and compared using the Mann-Whitney U test. Categorical variables were presented as counts (percentages) [n (%)] and compared using the χ² test.

The total sample was randomly divided into a training set and a validation set at a 7:3 ratio. The balance of baseline characteristics between the two sets was assessed using the t-test and χ² test (P > 0.05). In the training set, potential predictors with P < 0.05 in univariate analysis were initially screened. Subsequently, LASSO logistic regression (using the glmnet package with family=“binomial”, 10-fold cross-validation, and the λ-1se criterion) was employed for variable selection. The selected variables were then entered into multivariable logistic regression to identify independent predictors of rapidly progressive DR.

Of note, the same multivariable logistic regression method served two distinct purposes: first, to identify independent risk factors by estimating odds ratios (ORs) and 95% confidence intervals (CIs) (etiologic/associational analysis); and second, to serve as a benchmark prediction model for comparison with machine learning approaches (predictive/prognostic analysis).

For comparability across different modeling approaches, the same set of six predictors identified by multivariable logistic regression was used to construct Random Forest and Gradient Boosting Machine models, although these tree-based methods do not require pre-selection of variables. A sensitivity analysis using all candidate variables for Random Forest was performed to assess the impact of pre-selection. Model performance was compared using the area under the receiver operating characteristic curve (AUC) to determine the optimal model. Calibration was assessed using calibration curves based on deciles of predicted risk. Specifically, patients in the validation set were ranked by predicted probability and divided into 10 groups; within each group, the median predicted probability was plotted against the observed proportion of rapid progression (observed probability). LOESS smoothing was applied. Calibration was further quantified using the Brier score, calibration intercept, and the Hosmer-Lemeshow test. Clinical net benefit was evaluated with decision curve analysis (DCA). Model visualization and interpretability were achieved using nomograms and SHAP analysis. All tests were two-sided, and P < 0.05 was considered statistically significant. Given the class imbalance between rapid progression (n=66) and non-rapid progression (n=174) groups in the training set, we performed a sensitivity analysis using SMOTE (Synthetic Minority Over-sampling Technique) to oversample the minority class to achieve equal class sizes (n=174 each), then retrained the Random Forest model and compared validation AUC with the original model.

Transparency of the modeling pipeline: based on inclusion criterion (4) requiring complete baseline assessments, there were no missing values for any variable in this dataset; thus no imputation was performed. All continuous variables (age, diabetes duration, HbA1c, 24-hour urinary protein, GDF15, FAZ area, etc.) were Z-score standardized before model training to ensure comparability of contributions from variables with different units. Hyperparameters for Random Forest and Gradient Boosting Machine were tuned using grid search with 5-fold cross-validation: for Random Forest, the tuning ranges were number of trees (100, 300, 500), maximum depth (3, 5, 10), and minimum samples per node (5, 10, 20); for Gradient Boosting Machine, tuning ranges were learning rate (0.01, 0.05, 0.1), number of trees (100, 200, 300), and maximum depth (3, 5, 7). The parameter combination with the highest mean AUC from 5-fold cross-validation was selected for the final model. Regarding cross-validation, besides the 7:3 random split of the total sample into training and validation sets, 5-fold cross-validation was used within the training set during hyperparameter tuning, and the final model was evaluated on the independent validation set.

## Results

### Comparison of baseline characteristics between the training and validation sets

No statistically significant differences were observed between the training and validation sets across all evaluated baseline characteristics (*P* > 0.05), including demographic features (age, sex), diabetes-related history (diabetes duration, type of glucose-lowering medication, history of hypertension, smoking history, DRSS grade), anthropometric measurements (BMI), blood pressure (systolic blood pressure), HbA1c, renal function parameters (24-hour urinary protein excretion, eGFR, cystatin C), lipid metabolism markers (low-density lipoprotein cholesterol), electrolytes and trace elements (serum magnesium), vascular-related markers (plasma endothelin-1, Renin, Plexin B2), bone metabolism and growth factor-related markers (β-CTX, GDF15), and retinal imaging parameters (FAZ area). This indicates a balanced dataset partition, with good comparability of baseline characteristics between the training and validation sets, rendering them suitable for the subsequent development and validation of the risk stratification tool (**Table 1**).

**Table 1:** Comparison of baseline characteristics between patients in the training and validation sets.

Characteristic		Training set (n=240)	Validation set (n=102)	*t/χ²*	*P*
Age (years)		54.32 ± 11.20	53.78 ± 10.94	0.411	0.681
Sex	Male	118 (49.2)	50 (49.0)	0.001	0.980
	Female	122 (50.8)	52 (51.0)		
Diabetes duration (years)		8.45 ± 4.32	8.62 ± 4.51	0.328	0.743
SBP (mmHg)		135.62 ± 14.78	134.91 ± 15.21	0.403	0.687
BMI (kg/m²)		26.21 ± 3.89	26.52 ± 4.10	0.663	0.507
HbA1c (%)		8.15 ± 1.45	8.08 ± 1.52	0.402	0.687
Glucose-lowering medication class	Oral agents only	98 (40.8)	42 (41.2)	0.049	0.997
	Insulin only	58 (24.2)	24 (23.5)		
	Combination therapy	62 (25.8)	26 (25.5)		
	No medication	22 (9.2)	10 (9.8)		
History of hypertension	Yes	130 (54.2)	56 (54.9)	0.015	0.903
	No	110 (45.8)	46 (45.1)		
Smoking history	Never	120 (50.0)	51 (50.0)	0.009	0.995
	Past	48 (20.0)	20 (19.6)		
	Current	72 (30.0)	31 (30.4)		
24h urinary protein (g/24h)		0.23 ± 0.28	0.22 ± 0.26	0.309	0.758
eGFR (mL/min/1.73m²)		86.42 ± 18.51	85.90 ± 19.20	0.235	0.814
Cystatin C (mg/L)		1.02 ± 0.31	1.04 ± 0.33	0.535	0.593
LDL-C (mmol/L)		2.85 ± 0.96	2.79 ± 1.02	0.519	0.604
Serum magnesium (mmol/L)		0.84 ± 0.11	0.85 ± 0.12	0.748	0.455
Plasma endothelin-1 (pg/mL)		1.85 ± 0.78	1.79 ± 0.81	0.644	0.520
β-CTX(pg/mL)		412.35 ± 185.26	398.44 ± 192.13	0.621	0.535
GDF15(pg/mL)		1050.28 ± 450.36	1020.45 ± 470.21	0.548	0.584
Renin(pg/mL)		25.32 ± 12.11	24.81 ± 13.22	0.332	0.740
Plexin B2(pg/mL)		150.47 ± 60.23	145.62 ± 62.15	0.667	0.505
DRSS grade	No/Mild NPDR	120 (50.0)	51 (50.0)	0.019	0.995
	Moderate NPDR	72 (30.0)	31 (30.4)		
	Severe NPDR/PDR	48 (20.0)	20 (19.6)		
FAZ area (mm²)		0.31 ± 0.11	0.30 ± 0.10	0.803	0.423

### Univariate analysis of rapidly progressive diabetic retinopathy in patients with type 2 diabetes mellitus in the training set

In the training set of 240 patients with type 2 diabetes mellitus (T2DM), participants were categorized into a rapid progression group (n=66) and a non-rapid progression group (n=174) based on the occurrence of rapidly progressive diabetic retinopathy. Univariate analysis revealed that diabetes duration, HbA1c, 24-hour urinary protein quantification, GDF15, DRSS grade, and FAZ area were significantly associated with the occurrence of rapidly progressive diabetic retinopathy in patients with T2DM (*P* < 0.05). No statistically significant differences were observed between the two groups for the remaining demographic characteristics, blood pressure, body mass index, class of glucose-lowering medication, history of hypertension, smoking history, other renal function indicators, blood lipids, electrolytes, vascular-related indicators, or bone metabolism indicators (*P* > 0.05) (**Table 2**).

**Table 2:** Univariate analysis of factors associated with rapidly progressive diabetic retinopathy in patients with type 2 diabetes mellitus in the training set.

Characteristic		Rapid progression group (n=66)	Non-rapid progression group (n=174)	*t/χ²*	*P*
Age (years)		55.03 ± 11.25	54.12 ± 11.18	0.562	0.574
Sex	Male	34 (51.5)	84 (48.3)	0.015	0.902
	Female	32 (48.5)	90 (51.7)		
Diabetes duration (years)		10.61 ± 4.22	8.12 ± 4.28	4.039	0.001
SBP (mmHg)		137.45 ± 15.20	135.28 ± 14.75	1.009	0.314
BMI (kg/m²)		26.75 ± 4.05	26.18 ± 3.88	1.004	0.316
HbA1c (%)		8.68 ± 1.44	8.09 ± 1.47	2.792	0.005
Glucose-lowering medication class	Oral agents only	23 (34.9)	76 (43.7)	5.903	0.116
	Insulin only	18 (27.2)	38 (21.8)		
	Combination therapy	22 (33.3)	40 (23.0)		
	No medication	3 (4.6)	20 (11.5)		
History of hypertension	Yes	41 (62.1)	89 (51.1)	2.320	0.128
	No	25 (37.9)	85 (48.9)		
Smoking history	Never	33 (50.0)	87 (50.0)	0.007	0.997
	Past	13 (19.7)	35 (20.1)		
	Current	20 (30.3)	52 (29.9)		
24h urinary protein (g/24h)		0.42 ± 0.30	0.18 ± 0.24	6.440	0.001
eGFR (mL/min/1.73m²)		84.20 ± 19.10	86.95 ± 18.40	1.023	0.307
Cystatin C (mg/L)		1.07 ± 0.33	1.01 ± 0.30	1.345	0.179
LDL-C (mmol/L)		2.92 ± 1.01	2.84 ± 0.95	0.572	0.568
Serum magnesium (mmol/L)		0.83 ± 0.12	0.85 ± 0.11	1.226	0.221
Plasma endothelin-1 (pg/mL)		1.95 ± 0.81	1.83 ± 0.78	1.053	0.293
β-CTX(pg/mL)		390.40 ± 180.50	417.80 ± 187.30	1.022	0.307
GDF15(pg/mL)		1190.21 ± 490.51	1018.37 ± 448.62	2.581	0.010
Renin(pg/mL)		26.81 ± 12.94	25.08 ± 12.13	0.968	0.334
Plexin B2(pg/mL)		157.22 ± 63.13	148.57 ± 59.88	0.984	0.326
DRSS grade	No/Mild NPDR	20 (30.3)	100 (57.5)	16.385	0.001
	Moderate NPDR	24 (36.4)	48 (27.6)		
	Severe NPDR/PDR	22 (33.3)	26 (14.9)		
FAZ area (mm²)		0.36 ± 0.12	0.30 ± 0.10	3.922	0.001

### Multivariable logistic regression analysis for rapidly progressive diabetic retinopathy in patients with type 2 diabetes mellitus

Using the occurrence of rapidly progressive diabetic retinopathy as the dependent variable (1 = rapid progression group, 0 = non-rapid progression group), the six indicators that were statistically significant in the univariate analysis (diabetes duration, HbA1c, 24-hour urinary protein quantification, GDF15, DRSS grade, and FAZ area) were subsequently included in a Least Absolute Shrinkage and Selection Operator (LASSO) regression for variable selection (variable assignments are detailed in **Supplementary Table 1**). The optimal variables were selected using 10-fold cross-validation and the λ-1se criterion. All six predictive variables were retained and incorporated into the multivariable logistic regression model.

The multivariable logistic regression analysis (**Table 3**) demonstrated that diabetes duration, HbA1c, 24-hour urinary protein quantification, GDF15, DRSS grade, and FAZ area were independent risk factors for rapidly progressive diabetic retinopathy in patients with T2DM (all P<0.05). Their respective odds ratios (OR) and 95% confidence intervals (CI) were 1.156 (1.067-1.252), 1.286 (1.026-1.613), 1.897 (1.441-2.498), 1.282 (1.098-1.497), 1.908 (1.254-2.902), and 1.915 (1.514-2.423).

**Table 3:** Multivariable logistic regression analysis of rapidly progressive diabetic retinopathy in patients with type 2 diabetes mellitus in the training set.

Variable	β	SE	Wald	*P*	OR	95%CI
Diabetes duration	0.145	0.041	12.698	0.001	1.156	1.067~1.252
HbA1c	0.252	0.113	4.752	0.029	1.286	1.026~1.613
24-hour urinary protein	0.640	0.140	20.898	0.001	1.897	1.441~2.498
GDF15	0.249	0.079	9.811	0.006	1.282	1.098~1.497
DRSS grade	0.646	0.214	9.107	0.003	1.908	1.254~2.902
FAZ area	0.651	0.120	29.340	0.002	1.915	1.514~2.423

### Performance evaluation of machine learning models

Based on the key predictive variables identified through multivariable logistic regression—namely, diabetes duration, HbA1c, 24-hour urinary protein quantification, GDF15, DRSS grade, and FAZ area—three prediction models were constructed: Random Forest, multivariable logistic regression (serving as the benchmark), and Gradient Boosting Machine. This approach aimed to comprehensively evaluate and optimize the predictive performance for rapidly progressive diabetic retinopathy in patients with T2DM.

**Figure 1:**
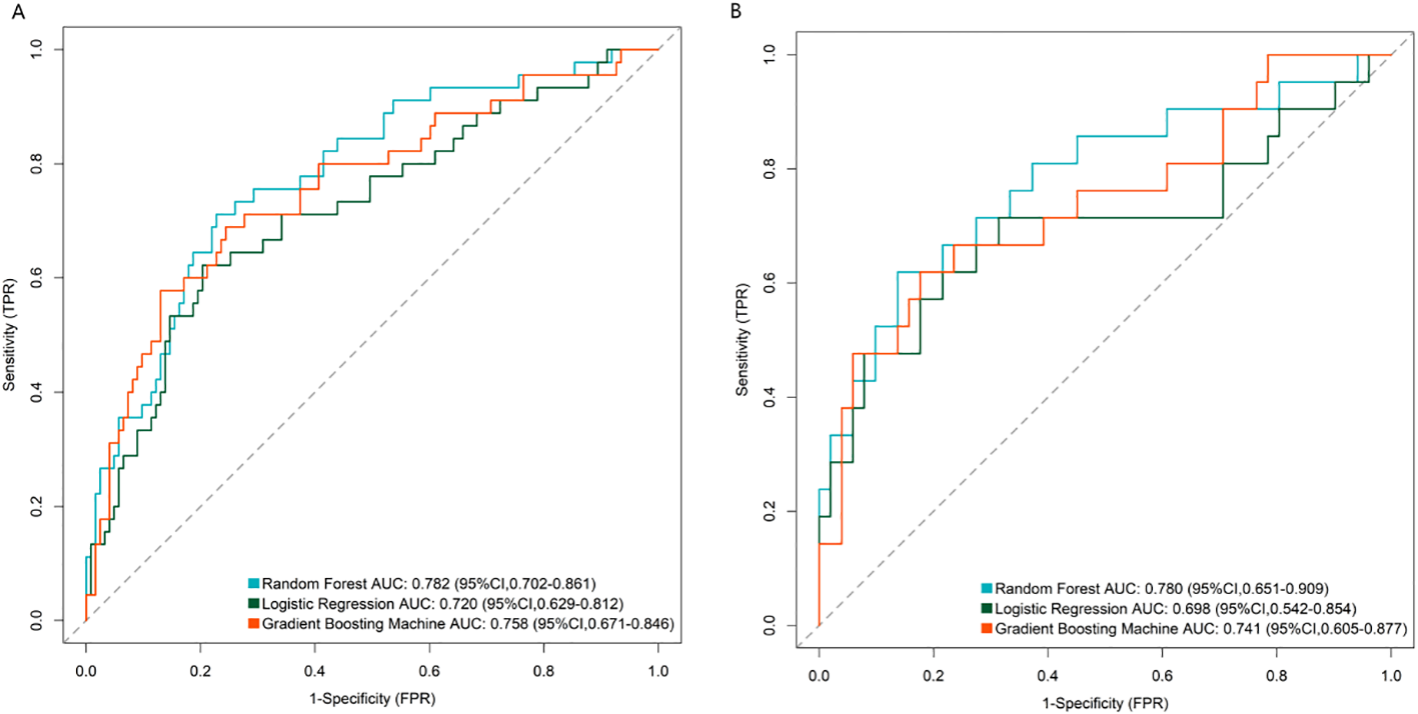
ROC curve analysis of the prediction model in the training **(A)** and validation **(B)** sets.

A systematic comparison of the models’ performance in the training and validation sets was conducted. In the training set, the area under the receiver operating characteristic curve (AUC) for the Random Forest model was 0.782 (95% CI: 0.702-0.861), compared to 0.758 (95% CI: 0.671-0.846) for Gradient Boosting Machine and 0.720 (95% CI: 0.629-0.812) for multivariable logistic regression. In the validation set, the Random Forest model achieved an AUC of 0.780 (95% CI: 0.651-0.909), whereas the Gradient Boosting Machine and multivariable logistic regression models achieved AUCs of 0.741 (95% CI: 0.605-0.877) and 0.698 (95% CI: 0.542-0.854), respectively. Although the Random Forest model achieved the numerically highest AUC in both datasets (validation AUC 0.780), DeLong test showed no statistically significant difference between Random Forest and Gradient Boosting Machine (AUC 0.741, P = 0.38). The difference between Random Forest and multivariable logistic regression (AUC 0.698) approached but did not reach statistical significance (P = 0.07). Therefore, both Random Forest and Gradient Boosting Machine demonstrated comparable discriminative ability, and Random Forest was not statistically superior. This result suggests that the Random Forest model possesses acceptable and relatively stable discriminatory capacity for rapidly progressive diabetic retinopathy in patients with T2DM, though it was not statistically superior to the Gradient Boosting Machine (**Figure 1**). Sensitivity analysis using all candidate variables to train the Random Forest model yielded a validation AUC of 0.776 (95% CI: 0.640-0.912), which was not significantly different from the original model (AUC 0.780, P = 0.82 by DeLong test), indicating that pre-selection did not compromise model performance. SMOTE sensitivity analysis: retraining the Random Forest model on the balanced training set yielded a validation AUC of 0.771 (95% CI: 0.639–0.903), compared with 0.780 (95% CI: 0.651–0.909) for the original model. The DeLong test showed no statistically significant difference (P = 0.58), suggesting that, despite the observed class imbalance, the main findings remained relatively stable.

**Figure 2:**
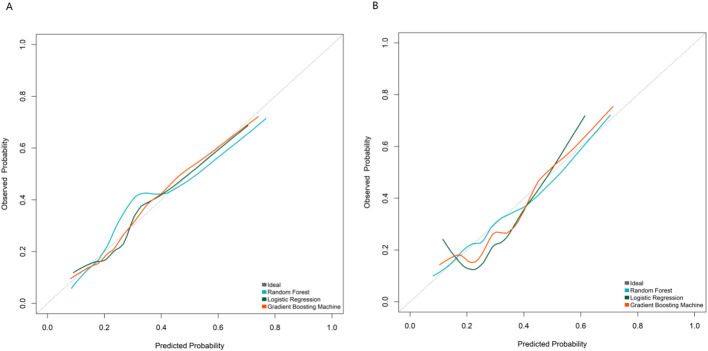
Curve analysis of the prediction model in the training **(A)** and validation **(B)** sets.

In the validation set at the default threshold (0.5), the Random Forest model achieved a sensitivity of 0.714 (95% CI: 0.512–0.861), specificity of 0.722 (95% CI: 0.612–0.814), precision of 0.625 (95% CI: 0.445–0.782), and F1-score of 0.667. Quantitative calibration metrics showed a Brier score of 0.189, a calibration intercept of -0.08 (95% CI: -0.31 to 0.15), and a Hosmer-Lemeshow test P-value of 0.42, all supporting good calibration. Furthermore, calibration curve analysis (**Figure 2**), constructed using deciles of predicted probability with LOESS smoothing as detailed in Methods, indicated a high degree of consistency between the predicted probabilities of the Random Forest model and the actual observed probabilities in both the training (**Figure 2A**) and validation (**Figure 2B**) sets, with the curves closely aligning with the ideal diagonal line. In contrast, while the calibration curves for the multivariable logistic regression and Gradient Boosting Machine models also showed good consistency, their fit was slightly inferior to that of the Random Forest model. These findings suggest that the Random Forest model is well-calibrated and yields more reliable predictions.

**Figure 3:**
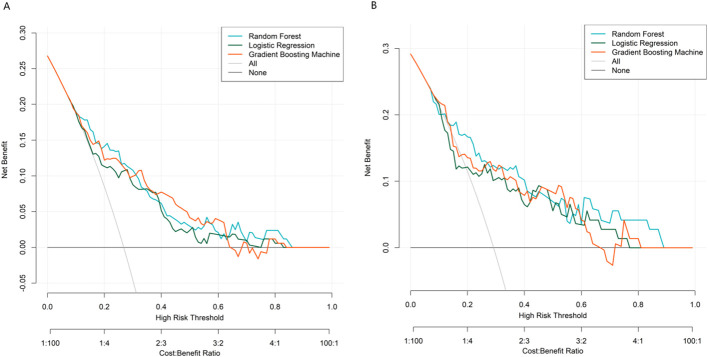
Clinical decision curve analysis of the prediction model in the training **(A)** and validation **(B)** sets.

Additionally, decision curve analysis (**Figure 3**) confirmed that across a wide range of high-risk threshold probabilities (0.1-0.8), applying the Random Forest prediction model resulted in a consistently higher net clinical benefit compared to the extreme strategies of intervening in all patients or in none. Its performance was also notably superior to that of the multivariable logistic regression and Gradient Boosting Machine models, highlighting its significant clinical utility.

In summary, the Random Forest prediction model developed in this study, based on clinical and laboratory indicators, exhibits reasonable predictive accuracy and satisfactory calibration, with potential clinical applicability, acknowledging that its performance was not statistically superior to the Gradient Boosting Machine. It provides an objective and reliable quantitative foundation for early risk stratification and the development of individualized intervention strategies for rapidly progressive diabetic retinopathy in patients with T2DM.

### Interpretability assessment of model predictions

**Figure 4:**
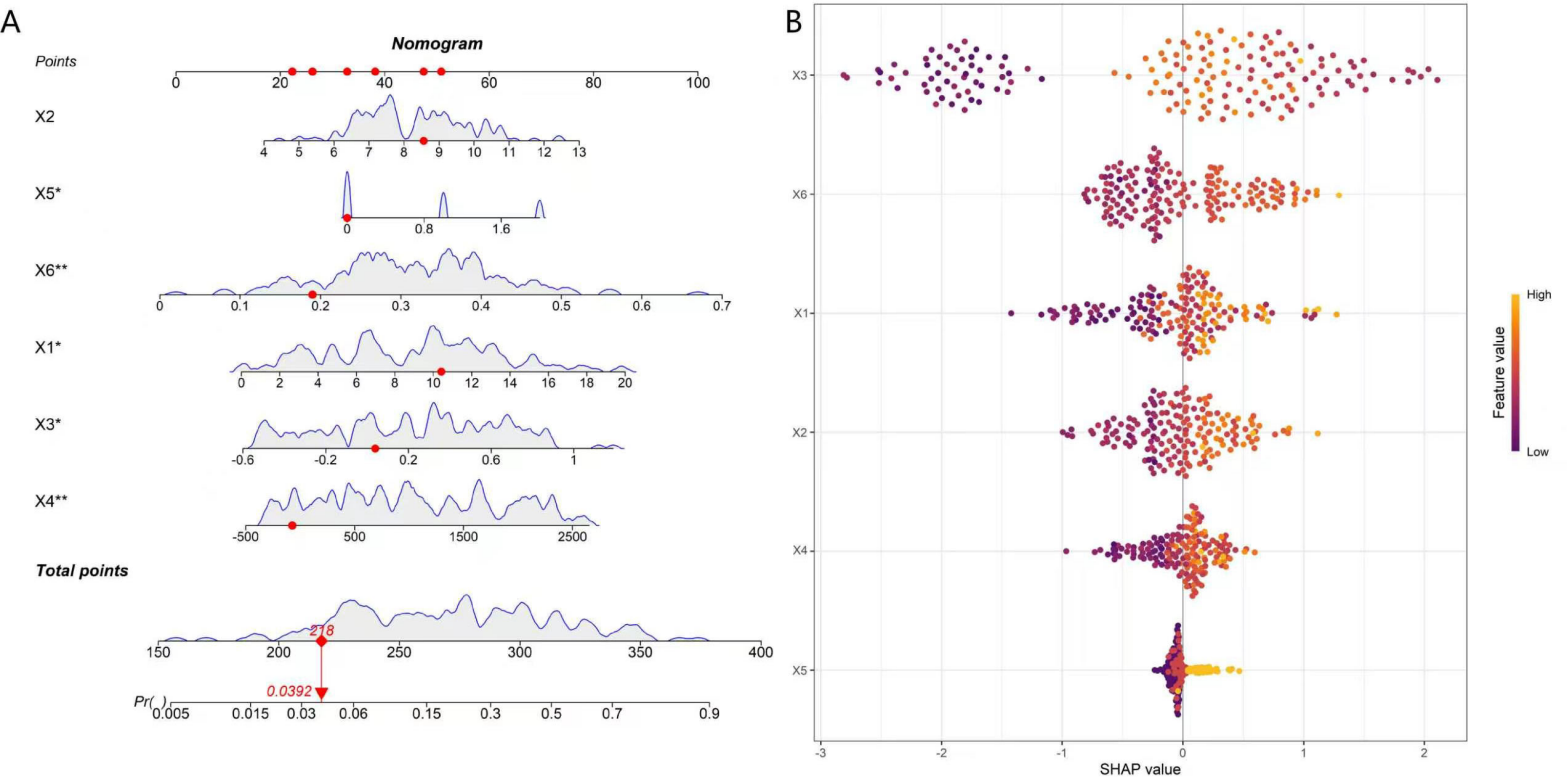
Model interpretability analysis. **(A)** Nomogram. **(B)** SHAP feature importance plot. X1, Diabetes duration; X2, HbA1c; X3, 24-hour urinary protein quantification; X4, GDF15; X5, DRSS grade; X6, FAZ area.

Utilizing the six core predictive variables identified by multivariable logistic regression (diabetes duration [X1], HbA1c [X2], 24-hour urinary protein quantification [X3], GDF15 [X4], DRSS grade [X5], and FAZ area [X6]), a nomogram model was constructed using the multivariable logistic regression algorithm to predict rapidly progressive diabetic retinopathy in patients with T2DM. As illustrated in **Figure 4**, this nomogram visually represents the contribution and direction of each clinical and laboratory indicator toward the probability of developing rapidly progressive diabetic retinopathy. The model results indicated that all six variables are risk promoters, with higher values for each indicator significantly increasing the predicted probability of rapid progression. The score distribution for each variable closely aligned with the trend of risk probability changes in the nomogram, clearly illustrating the quantitative contribution of different indicators to the model’s predictions.

SHAP (SHapley Additive exPlanations) analysis further quantified the relative importance of each feature, intuitively reflecting the direction and magnitude of the impact of feature values on the model’s predictions. In terms of feature importance ranking, 24-hour urinary protein quantification (X3) exhibited the widest distribution of SHAP values, signifying the greatest impact weight on the model’s output. This was followed by FAZ area (X6) and diabetes duration (X1), whose feature importances were next in line. The influence weights of HbA1c (X2), GDF15 (X4), and DRSS grade (X5) were relatively lower but remained crucial predictive characteristics of the model. Examining the color gradient of feature values (yellow for high values, purple for low values) revealed that high values for 24-hour urinary protein quantification, FAZ area, and diabetes duration corresponded to positive SHAP values. This further corroborates that elevations in these indicators substantially increase the model’s predicted probability of the “rapid progression” outcome, a finding entirely consistent with their identification as independent risk factors in the multivariable logistic regression analysis.

By combining the nomogram with SHAP analysis, this study not only developed a visualized tool for predicting individualized risk of rapidly progressive diabetic retinopathy but also clearly elucidated the decision-making mechanism of the Random Forest model in terms of feature importance and direction of impact. This effectively addresses the interpretability limitations often associated with the “black box” nature of traditional machine learning models. These results not only validate the clinical utility of the core predictive variables but also enhance the clinical interpretability and practical value of the prediction model, providing a visualizable and quantifiable theoretical and practical foundation for early risk identification and the formulation of individualized intervention strategies for patients with T2DM at risk for rapidly progressive diabetic retinopathy.

## Discussion

This study successfully developed and validated a risk stratification model for predicting rapid progression of diabetic retinopathy in patients with type 2 diabetes, utilizing multidimensional clinical, laboratory, and retinal imaging data. Through univariate and multivariable logistic regression analysis, six independent risk factors were identified: diabetes duration, hemoglobin A1c, 24-hour urinary protein excretion, growth differentiation factor 15, Diabetic Retinopathy Severity Scale grade, and foveal avascular zone area. Three machine learning models—random forest, multivariable logistic regression, and gradient boosting machine—were subsequently constructed. Among these, the random forest model achieved the numerically highest AUC in both the training (0.782) and validation (0.780) cohorts, but the difference from the gradient boosting machine was not statistically significant (DeLong test P = 0.38). Therefore, both random forest and gradient boosting machine demonstrated comparable discriminative ability, and neither was statistically superior. Furthermore, the incorporation of a nomogram and SHAP analysis enabled model visualization and interpretability by quantifying the contribution weight of each predictor, thereby providing a practical, quantifiable tool for clinical risk stratification of rapidly progressive diabetic retinopathy (14).

The six core predictors identified in this study encompass multiple dimensions—namely, glycemic control, renal impairment, inflammatory stress, baseline retinopathy severity, and macular microvascular structural alterations—collectively reflecting the complex pathophysiology underlying rapid diabetic retinopathy progression in type 2 diabetes (15). SHAP analysis revealed that 24-hour urinary protein excretion, foveal avascular zone area, and diabetes duration were the three most influential factors contributing to model prediction. Elevated 24-hour urinary protein excretion, a key marker of renal impairment, indicates the presence of diabetic nephropathy. Diabetic nephropathy and diabetic retinopathy frequently coexist as microvascular complications and mutually exacerbate disease progression. This finding aligns with previous studies identifying renal dysfunction as an independent risk factor for rapid diabetic retinopathy progression (16). The foveal avascular zone area reflects macular microvascular ischemia and structural disruption; its enlargement signifies aggravated local microcirculatory disturbance and serves as a critical warning signal for progression to proliferative diabetic retinopathy (17). Diabetes duration remains a classic risk factor for diabetic retinopathy progression, with longer exposure to hyperglycemia leading to cumulative microvascular damage and consequently elevating the risk of rapid progression (18).

Hemoglobin A1c, a core indicator of long-term glycemic control, is fundamentally implicated in the pathogenesis and progression of diabetic retinopathy. Its identification as an independent risk factor in this study further underscores the importance of intensive glycemic management in slowing diabetic retinopathy progression (19). Growth differentiation factor 15, a marker of metabolic stress and inflammation, is associated with systemic chronic inflammation and endothelial dysfunction. Its elevation may exacerbate microvascular inflammation and ischemia, thereby accelerating diabetic retinopathy progression, offering a novel perspective on the systemic inflammatory mechanisms involved in diabetic retinopathy (7). The Diabetic Retinopathy Severity Scale grade directly represents the severity of baseline retinal pathology; higher grades indicate more extensive microvascular damage. This factor serves as an intuitive clinical predictor of rapid progression, consistent with the widely recognized clinical observation that more severe baseline disease portends faster progression (20).

A key methodological strength of this study lies in the integration of traditional statistical approaches with modern machine learning techniques. Initially, univariate and multivariable logistic regression analyses were employed to identify and confirm independent risk factors, thereby ensuring clinical interpretability and statistical rigor. Subsequently, the random forest algorithm was utilized to capture complex nonlinear relationships among predictors, achieving comparable predictive accuracy to the gradient boosting machine model and numerically higher AUC than multivariable logistic regression, though the differences were not statistically significant. Concurrently, the nomogram translated the model into a visualized, individualized risk prediction tool. The integration of SHAP analysis elucidated the decision-making mechanism of the “black box” model, quantifying both the direction and magnitude of each predictor’s contribution to the outcome. This significantly enhances clinicians’ understanding of and trust in the model, establishing a foundation for its translation to bedside clinical application. Decision curve analysis further substantiated that applying this model confers greater net clinical benefit across a wide range of high-risk thresholds compared to “treat-all” or “treat-none” strategies, underscoring its substantial clinical utility.

Several limitations of this study should be acknowledged. First, as a single-center retrospective study, the generalizability of the model requires validation through multicenter, prospective external cohorts. We acknowledge this as a major limitation. To assess internal stability, we performed 100 random splits of the data (7:3 ratio) and retrained the Random Forest model on each training set; the AUC in the validation sets had a mean of 0.775 with a standard deviation of 0.04, indicating stable internal performance. However, external validation in independent cohorts from different populations and healthcare settings remains essential before clinical implementation (21). Second, the model incorporated only baseline static indicators and did not include dynamic data on glycemic control, renal function, or retinal status during follow-up, which may contain more critical predictive information regarding progression. Finally, the sample size was relatively limited, precluding detailed subgroup analyses based on age, diabetes type, or comorbidities, with an events-per-variable ratio of approximately 10 (66 events for 6 predictors), which is at the lower bound of recommended standards, and the dataset exhibited class imbalance (66 rapid progression vs. 174 non-rapid). Although our SMOTE sensitivity analysis confirmed the model’s robustness to this imbalance (validation AUC 0.771 vs. 0.780 for the original model, P = 0.58 by DeLong test), future studies with larger, more balanced multicenter cohorts or algorithms incorporating cost-sensitive learning are still needed to further validate the model. Additionally, although we pre-selected variables using LASSO before constructing Random Forest and GBM models, these models inherently perform feature selection. Our sensitivity analysis showed that using all candidate variables did not significantly change performance, suggesting that pre-selection was not detrimental but also not necessary. Future studies may directly input all variables into tree-based models. Additionally, the clinical availability of certain laboratory markers, such as growth differentiation factor 15, remains suboptimal, potentially hindering widespread model application. We also acknowledge that our conclusions regarding the model’s clinical utility are preliminary. Future prospective studies and external validations are needed to confirm its value in real-world settings.

From a practical perspective, this risk stratification model can be implemented for rapid risk assessment in outpatients with type 2 diabetes, enabling the identification of high-risk individuals for intensified retinal screening and early intervention, thereby facilitating targeted allocation of limited healthcare resources. From a policy standpoint, it is recommended to enhance the capacity for measuring core indicators—such as hemoglobin A1c and urinary protein excretion—in primary care settings, incorporate diabetic retinopathy risk stratification into chronic disease management protocols for diabetes, and optimize diabetic retinopathy prevention and control strategies. Regarding future research directions, multicenter prospective studies are necessary to validate model. Furthermore, efforts should focus on integrating dynamic monitoring data acquired during treatment and multi-omics biomarkers to develop refined models for distinct clinical subgroups. Ultimately, interventional studies guided by this model are warranted to confirm its value in improving patient visual outcomes.

This study successfully developed and validated a risk stratification model for rapid diabetic retinopathy progression in patients with type 2 diabetes, based on six core predictors: diabetes duration, hemoglobin A1c, 24-hour urinary protein excretion, growth differentiation factor 15, Diabetic Retinopathy Severity Scale grade, and foveal avascular zone area. The model exhibits reasonable discrimination, calibration, and clinical utility. It could serve as a complementary tool for individualized risk assessment, with both Random Forest and Gradient Boosting Machine showing acceptable performance, providing an objective, quantitative basis for the early identification of high-risk individuals and the optimization of intervention strategies.

## Data Availability

The original contributions presented in the study are included in the article/**Supplementary Material**. Further inquiries can be directed to the corresponding author.
