# Supplementary material for: Development of a risk stratification tool for rapidly progressive diabetic retinopathy in type 2 diabetes
Source: Front Endocrinol (Lausanne). 2026 Jun 19;17:1837957. doi: 10.3389/fendo.2026.1837957 (PMC13327953; doi:10.3389/fendo.2026.1837957)
Supplement: Supplementary file 1 [file Table1.docx]

**Supplemental Table 1.** Variable Assignments

| Variable | Meaning | Assignment |
| --- | --- | --- |
| X1 | Diabetes duration | Continuous variable |
| X2 | HbA1c | Continuous variable |
| X3 | 24-hour urinary protein | Continuous variable |
| X4 | GDF15 | Continuous variable |
| X5 | DRSS grade | 0=no/mild NPDR, 1=moderate NPDR, 2=severe NPDR/PDR |
| X6 | FAZ area | Continuous variable |
| Y | Outcome | 1=rapid progression group, 0=non-rapid progression group |
